# A comparative study of cell classifiers for image-based high-throughput screening

**DOI:** 10.1186/1471-2105-15-342

**Published:** 2014-10-21

**Authors:** Syed Saiden Abbas, Tjeerd MH Dijkstra, Tom Heskes

**Affiliations:** Institute for Computing and Information Sciences, Radboud University, Nijmegen, Netherlands; Department of Electrical Engineering, Eindhoven University of Technology, Eindhoven, Netherlands

## Abstract

**Background:**

Millions of cells are present in thousands of images created in high-throughput screening (HTS). Biologists could classify each of these cells into a phenotype by visual inspection. But in the presence of millions of cells this visual classification task becomes infeasible. Biologists train classification models on a few thousand visually classified example cells and iteratively improve the training data by visual inspection of the important misclassified phenotypes. Classification methods differ in performance and performance evaluation time. We present a comparative study of computational performance of gentle boosting, joint boosting CellProfiler Analyst (CPA), support vector machines (linear and radial basis function) and linear discriminant analysis (LDA) on two data sets of HT29 and HeLa cancer cells.

**Results:**

For the HT29 data set we find that gentle boosting, SVM (linear) and SVM (RBF) are close in performance but SVM (linear) is faster than gentle boosting and SVM (RBF). For the HT29 data set the average performance difference between SVM (RBF) and SVM (linear) is 0.42 *%*. For the HeLa data set we find that SVM (RBF) outperforms other classification methods and is on average 1.41 *%* better in performance than SVM (linear).

**Conclusions:**

Our study proposes SVM (linear) for iterative improvement of the training data and SVM (RBF) for the final classifier to classify all unlabeled cells in the whole data set.

**Electronic supplementary material:**

The online version of this article (doi:10.1186/1471-2105-15-342) contains supplementary material, which is available to authorized users.

## Background

The technology of high-throughput screening has facilitated many biological fields and has become a widely used method in drug discovery. It assists scientists in conducting millions of chemical as well as genetic tests to study biological paths. Cell biology is one of those fields which are currently focusing on analysis of massive amounts of cell image data produced by high-throughput screening [1–4]. Biologists study the morphology of these cells and can classify their phenotypes by visual inspection under a microscope. The microscopic study of a huge amount of cell image data has triggered the need for automatic methods to handle this huge amount of cell image data.

Machine learning and data mining have the potential to objectively and effectively analyze the massive amounts of image data [5]. In recent years, many studies have shown advantages of using classification methods to classify images based on features derived from them [2, 6–11]. Examples of classification methods are the Support Vector Machine (SVM), the gentle boosting classifier, Linear Discriminant Analysis (LDA), the K-nearest neighbor (KNN) classifier, the multi-layered perceptron, Artificial Neural Networks (ANNs) and the decision tree classifier [11–16].

Usually, there are three steps involved in classification of cells as shown in Figure one by Jones et al. [7]. The first step is segmentation and feature calculation. The second step concerns the training of classification models on a training set and their performance evaluation with cross-validation. The training set is a subset of a few thousand cells visually classified by a biologist. The third step boils down to the classification of whole screen using the best performing classifier from step 2.

Typically, the second step is performed many times in an iterative feedback and machine learning approach as proposed in [7]. In this approach, biologists classify a number of cells, then train the classifier and inspect the classified cells. If the classification method gives high error on some of the important phenotypes, the biologists classify more cells of those phenotypes and again train the classifier. Thus, segmentation, feature calculation, and phenotype classification of all images (including unlabeled) are done only once whereas classifier training is done many times. Biologists are therefore helped by classification methods that are fast and give high performance. Table 1 gives an overview of software packages that are commonly used for processing of images obtained in high-throughput screening. There are numerous software packages available for cell detection, feature extraction and feature analysis [2, 5, 6]. These software tools identify cells from images and measure features of identified cells. Different classification methods are used by these software packages as shown in Table 1.

**Table 1:** **Open source tools for high-throughput screening**

Tool [17]	Language	Classifier	Advantage
WND-CHARM [18]	C++	Weighted Nearest Neighbor	Many image features
Enhanced CellClassifier [19]	Matlab	SVM	Good classifier
FARSIGHT [20]	C++	Supervised Spectral Clustering	Programmer friendly
CellMorph, EBImage [21]	R	SVM	Link to machine
			learning algorithms
CellCognition [22]	Python	Hidden Markov Model	Classifies movies
CellXpress [23]	C++	R package for SVM	Phenotypic profiling
Ilastik [24]	Python	Random Forest	Interactive segmentation
BIOCAT [6]	Java	Nearest Neighbor, Random Forest,	User friendly and extensible
		SVM and Decision Trees	

To the best of our knowledge, there is no study that compares the performance of different classification methods and their suitability in an iterative feedback and machine learning setting for high-throughput screening of images. In this paper we compare classification methods based on accuracy and cross-validation time. We also explore how performance and computational time vary with a different number of phenotypes. We use two data sets of HT29 and HeLa cancer cells that have different numbers of features and phenotypes. We investigate which classifier is a good choice in terms of performance and cross-validation time. Cross-validation time is important because it is the time needed to evaluate the performance of a classifier and cross-validation needs to be done many times in training a classifier in an iterative fashion. The next part describes the data sets, the classification methods and the approach used in this study. The last part consists of results and discussion.

## Method

### Data description

For this study, we used two data sets. The first data set contains HT29 colon cancer cells which was first published by Moffat [3] and is available as image set *BBBC*018*v*1 from the Broad Bioimage Benchmark Collection [25]. Cells were stained for DNA, actin and phospho-histone proteins. DNA was stained with Hoechst 33342 fluorescent dye. Actin proteins were stained with a fluorescent phalloidins dye while phospho-histone proteins were stained with a fluorescent tagged antibody [3]. Carpenter et al. [17] developed the open source software package CellProfiler through which they identified about 8.3 million cells in 40,000 images of the HT29 data set. Each cell has a set of 615 features which are shape, intensity and texture features of the DNA, actin and phospho-histone (ph3) channels. These features consist of geometric (extension, eccentricity, axis lengths, size and size ratio between cell and nucleus etc.), Haralick (angular moments, contrast, correlation, variance and entropy etc.) and Zernike features. The HT29 data set contains linearly dependent features because some features were derived from other features. This linear dependency poses no problem for the SVM and boosting classifiers, but is problematic for standardLDA.

Figure one in Jones et al. [7] summarizes the cell identification and measurement of cell features for the HT29 data set. A subset of cells was presented to biologists who classified the cells into one of 14 phenotypes (listed in Table 2). Figures three and four in Jones et al. [7] show a total of 2581 positive and 13,139 negative examples of 14 cell phenotypes. A cell is a positive example if it has a particular phenotype and it is a negative example if not. In this study, we only used the positive examples. We found 55 cells that had two phenotypes associated with them and removed these ambiguously classified cells. For example, there were two cells labeled both as actin blebs (AB) and crescent nuclei (CN). There were 2526 cells left after removing the ambiguously classified cells. Table 2 shows the 14 phenotypes with the number of cells for each of the phenotypes.

**Table 2:** **HT29 colon cancer cells with 14 phenotypes**

Phenotypes	Cells
Actin blebs (AB)	107
Actin dots (AD)	111
Anaphase -Telophase (AT)	182
Angular cell edges (ACE)	73
Crecent nuclei (CN)	185
Large spread cells (LSC)	201
Long projections (LP)	59
Metaphase (MP)	563
Motile (M)	190
Peas in a pod (PIP)	34
Perpheral actin (PA)	59
Phospho-Histone H3 dots (PHD)	264
Prometaphase (PMP)	345
Prophase (PP)	153
Total	2526

Each cell has 615 features.

The second data set contains HeLa cancer cells which was created by Fuchs et al. [8] for testing the EBImage software package. The cells were stained for DNA, actin and tubulin. The data set contains a total of 2545 cells with 51 features for each cell. These 51 features consist of geometric, Haralick and Zernike features calculated from the intensity and textures of a cell using the EBImage package [21] as shown in Figure one (E,F) of Fuchs [8]. There are 10 phenotypes as shown in Table 3.

**Table 3:** **HeLa cancer cells with 10 phenotypes**

Phenotypes	Cells
Actin fiber (AF)	170
Big cells (BC)	310
Condensed cells (C)	338
Debris (D)	219
Lamellipodia (LA)	258
Metaphase (MP)	186
Membrane blebbing (MB)	110
Normal cells (N)	542
Protrusion and elongation (P)	315
Telophase (Z)	97
Total	2545

Each cell has 51 features.

Tables 2 and 3 show that each phenotype is represented by a different number of cells which makes the data sets class imbalanced. The HT29 data set suffers from greater class imbalance than the HeLa data set. In case of the HT29 data set, the phenotype with the largest number of cells (metaphase) is about 16 times bigger than the phenotype with the smallest number of cells (peas in a pod), while in the case of the HeLa cells the phenotype with the largest number of cells (normal cells) is about 5 times bigger than the phenotype with the smallest number of cells (telophase). To make sure that the relative frequencies among phenotypes remain roughly the same across all folds, we used 20-fold cross-validation with stratified sampling on the class variables.

### Classification methods

There is no single classification method which outperforms all other classification methods on all data sets. The list of classification methods is large and every method has its own strengths and limitations [12, 13]. In this study we include five classification methods: SVM (RBF), SVM (linear), gentle boosting, joint boosting (CPA) and LDA. We choose SVM (RBF), because it has been used in [8, 26] to classify the HeLa data set. Joint boosting (CPA) is included since it is part of the CellProfiler Analyst software applied in [7] to analyze the HT29 data set. The other three classifiers are included to check whether we can obtain similar performance with simpler classifiers. We include gentle boosting as a lean alternative to joint boosting (CPA) and SVM (linear) as an alternative to SVM (RBF). We include LDA because it is traditionally considered to be a good benchmark classifier. The details of the implementation and tuning of the parameters of the classifiers are as follows.

- Joint boosting (CPA): A multi-class version of gentle boosting with shared regression stumps [15]. This classifier learns to use common features shared across the phenotypes. The classifiers for each phenotype are trained jointly, rather than independently [15]. CellProfiler Analyst (CPA) has implemented the idea of [15] without sharing features. In boosting, the classifiers are built using regression stumps. The learning time increases with increasing number of regression stumps. The manual of CellProfiler Analyst advises the use of 50 regression stumps and [7] has also used 50 regression stumps for the HT29 data set. In this study we also use 50 regression stumps for joint boosting (CPA). Since, as we will see below, the performance of joint boosting (CPA) with the recommended 50 regression stumps falls short, we also considered using the same method with 200 regression stumps. We will refer to those as joint boosting (CPA-50) and joint boosting (CPA-200). For joint boosting (CPA), we used CellProfiler Analyst 2.0 (*r*11710). This method uses the one-versus-all strategy for multi-class classification.
- Gentle boosting: Boosting methods such asadaboost, real-adaboost, logit-boost and gentle boost perform well on images or scenes cluttered with objects [15, 27, 28]. Boosting methods build a good classifier from many weak classifiers and are similar to decision trees in building classification rules [15, 28]. We use 50 regression stumps for gentle boosting. This method uses the one-versus-all strategy for multi-class classification and also uses multiple features with different thresholds and different weights for each phenotype [27, 28].
- Support vector machine with radial basis function (RBF): Generally, the SVM (RBF) classifier is better in performance and is tolerant to irrelevant and interdependent features as compared to decision trees, neural networks and K-nearest neighbor classifiers [9, 12, 13, 29, 30]. SVM (RBF) is a useful method when data is not linearly separable but is slower because of the optimization of the hyper parameters *C* and *γ*. The hyper parameter *C* is the cost parameter which gives a trade-off between training error and model complexity [31, 32]. The higher the value of the *C*, the higher cost for non-separable examples [31]. The hyper parameter *γ* is the inverse of the width of the radial basis function. For selection of parameters *C* and *γ*, a grid search was performed on values *C*∈ [ 2^-1^,2^0^,…,2^6^] and *γ*∈ [ 2^-5^,2^-4^,…,2^1^] for both data sets. A 5-fold cross-validation was performed to select the hyper parameters. In this study, the LIBSVM 3.17 library [30] is used which implements the one-against-one strategy for multiclass classification.
- Linear support vector machine (SVM linear): SVM (linear) is an alternative to SVM (RBF) for large data sets where with/without nonlinear mappings gives similar performance [12, 33]. SVM (linear) requires only one hyper parameter *C* which reduces the training and testing times. A 5-fold cross-validation was performed to select the hyper parameter. The search for the optimal hyper parameter *C* was performed on values *C*∈ [ 2^-5^,2^-4^,…,2^6^] for both data sets. In this study we used the Liblinear 1.94 library [33] which uses a one-vs-all approach for multiclass classification. This library has different versions of regularized linear classification. We used the *L*_2_ regularized linear classification with the *L*_2_ loss function because it is computationally fast. The performance was similar for the other loss functions.
- Linear discriminant analysis (LDA): LDA is a useful method when features are linearly independent and normally distributed. LDA tries to maximize the separation between classes by estimating classes boundedness as a linear combination of the features. LDA does not require any parameter tuning. As the HT29 data set contained linearly dependent features, we used the Moore-Penrose pseudo inverse for the covariance matrix which is provided in the Matlab implementation of LDA.

For performance evaluation of each classifier 20-fold cross-validation was performed. The performance (accuracy) of a classifier is defined as the number of correctly classified cells divided by the total numbers of the cells. For SVM (RBF), SVM (linear), gentle boosting and LDA classifiers, the time elapsed by the 20-fold cross-validation was recorded by using the *tic*/*toc* functions available in Matlab. The *tic*/*toc* functions resemble the wall-clock time. The cross-validation time also includes the time of the tuning of the parameters required by a classifier. The implementation of joint boosting (CPA) is in Python while other classifiers are implemented in C++ and called from Matlab using wrapper functions. The Python implementation of joint boosting uses the *time* function which is similar to the *tic*/*toc* functions of Matlab. Features of both data sets were normalized and then scaled between 0 and 1. The analysis was performed on a Macbook Pro, Intel core i5 CPU with 2.4 GHz processing speed using Matlab version *R*2013*a* installed on OS X 10.9.3 (13D65).

### Approach

**Figure 1:**
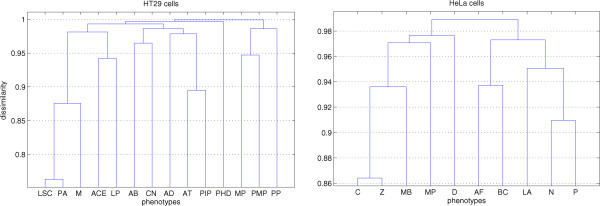
**Dendrograms of merging of phenotypes based upon the dissimilarity matrix obtained from the averaged confusion matrix of four classifiers: gentle boosting, SVM (linear), SVM (RBF) and LDA.**

To find out how the performance and computational complexity of the classification methods varies with the number of phenotypes, we constructed smaller numbers of phenotypes by merging the most confused phenotypes. First we carried out an analysis by using the SVM (Linear), SVM (RBF), gentle boosting and LDA classifiers with all 14 and 10 phenotypes of HT29 and Hela cells respectively. For each of the data sets, the four confusion matrices obtained from each classifier were averaged (seeAdditional file 1). We added the upper and lower triangular parts of the averaged matrix to obtain a symmetric matrix of the total confusions among phenotypes. Each row of the symmetric matrix was divided by the sum of that row to a get a normalized symmetric matrix for each of the data sets. These normalized symmetric matrices were converted into dissimilarity matrices by subtracting from one.We performed hierarchical clustering with the unweighted average distance (UPGMA) method to merge phenotypes. Figure 1 shows the dendrograms obtained as a result of clustering. For example, the phenotypes LSC and PA in case of HT29 cells are most similar as shown in the dendrogram in the left panel of Figure 1. We merged these two phenotypes and labeled them as one phenotype. After merging, we were left with 13 phenotypes for HT29 cells on which we performed the analysis using all classifiers. Then, we again merged the next two most similarphenotypes which were the new merged phenotype obtained in the last merging and M, as shown in the dendrogram of HT29 cells in the left panel of Figure 1. This process of merging and analysis continued until we were left with only two phenotypes. The merging of the phenotypes was the same for all of the classifiers. We did not employ joint boosting (CPA) in constructing of the dissimilarity matrix because CellProfiler Analyst (CPA) does not provide easy access to the confusion matrix. For joint boosting (CPA), we used the same fixed merging of phenotypes which was obtained from the other four classifiers (Figure 1).

## Results and discussion

### Results

**Figure 2:**
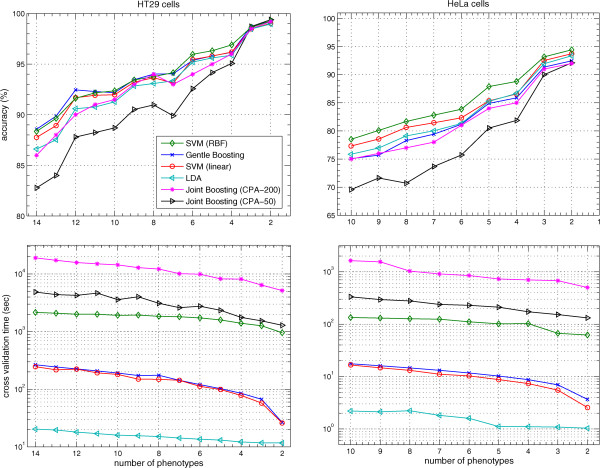
**A comparison of performance and cross-validation time with all features of the HT29 and HeLa data sets.**

Figure 2 shows that the performance of classification methods increases with a decrease in the number of phenotypes for both data sets. In case of 14 phenotypes of HT29 cells, accuracies of SVM (RBF), SVM (linear), gentle boosting, joint boosting (CPA-50) and LDA are 88.4*%*, 87.8*%*, 88.6*%*, 82*%* and 86.6*%* respectively. For HT29 cells, there is no noticeable difference in the performance among SVM (linear), SVM (RBF) and gentle boosting. LDA is slightly worse than SVM (linear), SVM (RBF) and gentle boosting in case of more than 7 phenotypes. Joint boosting (CPA-50) suffers from lower performance except for two and three phenotypes. In case of 10 phenotypes of HeLa cells, accuracies of SVM (RBF), SVM (linear), gentle boosting, joint boosting (CPA-50) and LDA are 78.5*%*, 77.3*%*, 75*%*, 69.8*%* and 75.9*%* respectively. For HeLa cells SVM (RBF) outperforms the other classifiers while there is no noticeable difference in performance among SVM (linear), gentle boosting and LDA classifiers as shown in the upper right panel of Figure 2. Previously, HeLa cells were classified with SVM (RBF) by Fuchs et al. [8] in which the performance was about 78 *%* for 10 phenotypes which is about the same as SVM (RBF) in our analysis. Joint boosting (CPA-50) is the worst in performance on HeLa cells compared with the other classifiers.Cross-validation is computationally intensive depending upon the number of parameters that need tuning, the number of cells, the number of features and the number of folds of the cross-validation. The lower left and right panels of Figure 2 show the time of 20-fold cross-validation for the HT29 and HeLa cells respectively. The cross-validation times of the SVM (linear) and SVM (RBF) include the learning time of the hyper parameters. The cross-validation time increases with the number of phenotypes as shown in Figure 2. Gentle boosting, joint boosting (CPA-50), SVM (linear), SVM (RBF) and LDA took on average 265, 4892, 246, 2155 and 20 seconds respectively for 20-fold cross-validation with 14 phenotypes on HT29 cells as shown in the lower left panel of the Figure 2. In case of HeLa cells, the time taken by 20-fold cross-validation with 10 phenotypes was 16, 334, 17, 134 and 2 seconds for gentle boosting, joint boosting (CPA-50), SVM (linear), SVM (RBF) and LDA respectively as shown in the lower right panel of the Figure 2.

To put the cross-validation time in perspective, we timed the calculation for (1) image segmentation and feature extraction and (2) the time to label all cells in a screen. The software packages and data related to the HeLa data set are available on [34]. We took the data from this site and reran it to find the time taken by segmentation and feature measurements. It took about 4321 seconds to segment and calculate features of 32778 cells in 516 images. Each image size was 670×510 pixels. Since we had unlabeled data of the HeLa data set, we trained the classifiers with optimal parameters obtained through cross-validation and noted the time used by the classifiers to label all unlabeled data. On about 1.6 million cells, it took about 7, 11, 20 and 324 seconds by gentle boosting, SVM (linear), LDA and SVM (RBF) respectively.

Joint boosting (CPA-50) has the worst performance of all classifiers under consideration. To find an explanation for the bad performance of joint boosting (CPA-50), we increased the number of regression stumps from 50, as used by [7] and advised by the CellProfiler manual, to 200. In case of 14 phenotypes of HT29 cells, joint boosting (CPA) with 200 regression stumps gives an accuracy of 86*%* in 19047 seconds. In case of 10 phenotypes of HeLa cells, joint boosting (CPA) with 200 regression stumps reaches an accuracy of 75*%* in 1631 seconds. We tried even more regression stumps, but did not find any further substantial performance improvement. In any case, by increasing the number of regression stumps, the accuracy of joint boosting (CPA) does become close to the other classifiers as shown by the line for joint boosting (CPA-200) in Figure 2. The increase in number of regression stumps increases the performance evaluation time considerably and makes joint boosting (CPA) an order of magnitude slower than its competitors.

LDA is the fastest among all classifiers in cross-validation but suffers from low performance especially in case of more than seven phenotypes. Cross-validation time is the same for SVM (linear) and gentle boosting, but gentle boosting suffers from lower performance in the case of the HeLa data set as shown in Figure 2. For the HT29 data set, SVM (linear) has an overall similar performance as compared to SVM (RBF) and gentle boosting. SVM (RBF) is a slow method which consumes time in a grid search of hyper parameters and there is little performance gain over other classifiers in the case of HT29 cells. For HT29 cells, the average performance difference between SVM (RBF) and SVM (linear) is 0.42*%*. On average across all number of phenotypes SVM (linear) is about 15 times faster than SVM (RBF) in the case of HT29 data set. For HeLa cells, SVM (RBF) is slower than SVM (linear), gentle boosting and LDA, but has better performance. For HeLa cells, the average difference in performance between SVM (RBF) and SVM (linear) is 1.41*%*. On average across all number of phenotypes SVM (linear) is about 12 times faster than SVM (RBF) in the case of HeLa data set.

**Figure 3:**
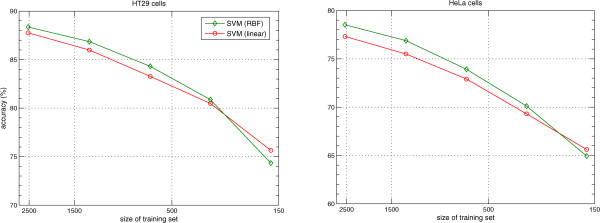
**A comparison of performance of SVM (RBF) and SVM (linear) on HT29 and HeLa data sets with different sizes of training sets.**

Results in Figure 2 suggest that SVM classifiers overall give better performance on both data sets with different number of phenotypes. To find how accuracy depends on the number of cells for the SVM classifiers, we subsampled data sets by (1/2, 1/4, 1/8, 1/16) using stratified sampling on phenotypes. We used stratified sampling meaning that each subsampled data set had the same fraction of cells of each phenotype as the full data set. We randomly selected 50 times cells in each of these subsamples and performed 20-fold cross-validation on the selected cells. Both data sets show a decrease in performance with a decrease in number of cells as shown in Figure 3. For HT29 cells, the average performance difference between SVM (RBF) and SVM (linear) is 0.84*%* across different sizes of training sets. For HeLa cells, SVM (RBF) has a performance gain of 1*%* over SVM (linear) across different sizes of training sets. Interestingly, performance differences are smaller for the subset of 1/8 and SVM (linear) has a small advantage for the subset of 1/16.

Our study finds that the difference in performance is small between SVM (linear) and SVM (RBF) but that SVM (linear) is faster than SVM (RBF) on both data sets. This finding leads us to investigate further which of these two classifiers is suitable in the iterative approach of training classifiers and their performance evaluation using cross validation. To answer this question, we investigated whether the misclassified cells by SVM (RBF) are a subset of the misclassified cells by SVM (linear). We ran 100 times 20-fold cross-validation on both data sets. We call a cell misclassified if in 80 or more of the 100 runs it was wrongly classified. For the HT29 data set, we find that 75*%* of the cells misclassified by SVM (RBF) are also misclassified by SVM (linear). For HeLa data set, we find that 87*%* of the cells misclassified by SVM (RBF) are also misclassified by SVM (linear). Since the fraction of cells misclassified only by SVM (RBF) is relatively small, this suggests that it is safe to use the faster classifier in the iterative improvement of the classifier. Once biologists are satisfied with the labeled phenotypes of the training data and classifier, they can use SVM (RBF) to classify all unlabeled cells in whole data set. In this approach, the iterative phase would be fast with SVM (linear) and final labeling (testing phase) would have the performance gain with SVM (RBF).

**Figure 4:**
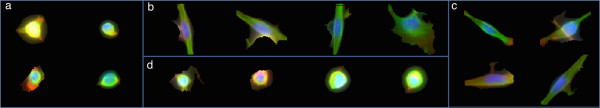
**Misclassification of condensed (C) cells and protrusion-elongation (P) cells by SVM (RBF). (a)** Correctly classified condensed cells **(b)** Condensed cells misclassified as protrusion-elongation cells **(c)** Correctly classified protrusion-elongation cells **(d)** Protrusion-elongation cells misclassified as condensed cells.

In Figure 4, panel (a) and (c) show exemplary cells of the condensed (C) and protrusion-elongation (P) phenotypes from the HeLa data set. Panel (b) in Figure 4 shows some of the cells labeled as condensed cells but looking like protrusion-elongation cells and always classified as the protrusion-elongation phenotype by the SVM classifiers. Similarly, some of the cells labeled as protrusion-elongation cells look like condensed cells and are always classified as the condensed cells by the SVM classifiers as shown in panel (d) of Figure 4. This figure can be compared with Figure 1(E) of [8]. Perhaps, the cells in panels (b) and (d) are accidentally labeled incorrectly.

**Figure 5:**
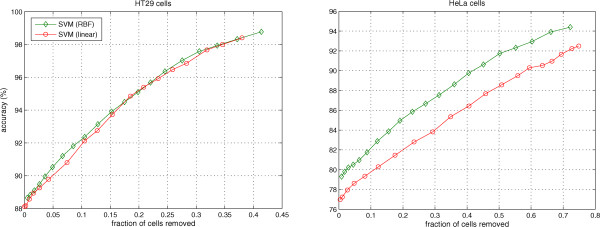
**Change in performance of SVM (linear) and SVM (RBF) for HT29 and HeLa data sets by removing cells with lower posterior probabilities of phenotypes.**

Sometimes, biologists focus attention on the “good” or more “prototypical” cells when evaluating a certain feature. Thus, the idea of dropping the difficult to classify cells and only focusing on more prototypical cells would be helpful for biologists in studying a certain phenotype. We explore the trade-off between the number of cells included (not dropped) and classification accuracy. The posterior probability of the phenotype of each cell provides a measure of certainty provided by classifiers [30, 33, 35]. By thresholding the posterior probability, we exclude cells that the classifier considers close to the decision boundary and explore the trade-off between the fraction of cells included and the accuracy. We used only the SVM based classifiers for posterior probability estimates because these classifiers are good choices assuggested by the performance results. The LIBSVM implementation of SVM (RBF) applies the sigmoid function described in [36, 37] to estimate posterior probabilities as a post processing step. We applied the same post processing step to obtain the posterior probabilities for SVM (linear). We drop those cells for which the maximum posterior probability over the phenotypes is lower than a particular threshold (plotted on the x-axis in Figure 5). Figure 5 shows the results obtained by thresholding of the posterior probabilities from the HT29 and HeLa data sets with 14 and 10 phenotypes respectively. It reveals an increase in the accuracy of SVM (linear) and SVM (RBF) for both data sets. We increased the probability threshold to that value where all cells of a certain phenotype become excluded. Thus, the increase in performance is not due to all cells of a phenotype being removed. These results suggest that biologists can use the posterior probabilities to focus only on more prototypical cells while studying features of phenotypes of cells.

### Discussion

Several other studies have evaluated classification performance based on images obtained in high-throughput screening [4, 9, 10, 12, 38, 39]. Classification methods are mostly applied for the classification of sub-cellular protein localization, cell phase, cell phenotype and cellular compounds on data sets obtained in high-throughput screening [12, 39]. Previous studies have applied different methods for classification of different number of phenotypes with different number of features [9, 10, 12, 38, 39]. The geometric, Haralick and Zernike features are the most commonly used features for image-based high-throughput screening of cells in different software packages, but with different segmentation, feature selection and classification methods [5, 6, 24]. Our study recommends software packages to include both SVM (linear) and SVM (RBF) classifiers to help biologists in performing a fast and efficient analysis of high-throughput data.

We imagine a partition of labor of analyzing a high-throughput screen in three steps as presented by Jones et al. (2009) in Figure 1 [7]. The first step consists of image segmentation and feature calculation. This is a computation intensive step and took about 72 minutes for a subset of the HeLa data set consisting of 516 images of 670 by 510 pixels with 232K cells. While computation intensive, this step typically does not involve much manual labor. An investigator can try several image segmentation algorithms and judge the quality of the segmentation. Importantly, this step is independent of later steps.

The second step involves iterative training of a classifier. Here an investigator is presented with a set of randomly selected images and the investigator provides the phenotypes (labels) to the computer. From this initial set, the classifier is trained and its performance (accuracy) is computed with cross validation. This performance is evaluated by the investigator who can then decide to label more cells either randomly selected by the computer or selected from certain phenotypes in which the investigator is interested. Either way, as this iterative training of the classifier might be done many times, the classification algorithm should be relatively fast, possibly at the expense of a reduction of testing accuracy. As we have shown SVM (linear) to be 13 times faster than SVM (RBF) at the expense of a reduction in accuracy of 0.9*%* (average over both data sets and all number of phenotypes), we propose the use of SVM (linear) for this second step.

The third step is classification of the phenotypes of all cells in the screen. Given its small but clear classification accuracy benefit, we advocate the use of SVM (RBF) as others [8–10, 26, 38]. As an extension, we investigated whether a classifier’s notion of its own classification accuracy as the posterior probabilities can be used to screen for “high quality” cells. Indeed, as we show in Figure 5, thresholding the posterior probabilities improves the objective accuracy. Thus, in case an investigator has the luxury of a large number of cells of a particular phenotype in a particular experimental condition, he or she can decide to focus on the cells that have the particular phenotype with more certainty.

We did not draw any conclusion from the similarities among phenotypes shown in Figure 1. Some previous studies find cell-to-cell variations among cells of the same phenotype [40]. In future studies it would be interesting to explore the performance of more classification methods on other image-based high-throughput data sets with more focus on the similarities between phenotypes and the cell-to-cell variations among cells of the same phenotype.

## Conclusion

In summary, our study advocates that among the considered classifiers and data sets in this study, SVM (linear) is the appropriate choice for high-throughput screening data sets in iterative training of the classifier while SVM (RBF) is the appropriate choice for the final classifier to classify all cells including unlabeled cells.

## Electronic supplementary material

Additional file 1: **Contains confusion matrices obtained from each of the classifiers for both data sets.** These matrices were used in creating hierarchical clusters shown in Figure 1. (PDF 55 KB)
