# Supplementary material for: A comparative study of cell classifiers for image-based high-throughput screening
Source: BMC Bioinformatics. 2014 Oct 21;15(1):342. doi: 10.1186/1471-2105-15-342 (PMC4287552; doi:10.1186/1471-2105-15-342)
Supplement: Supplementary file 1 — Additional file 1: Contains confusion matrices obtained from each of the classifiers for both data sets. These matrices were used in creating hierarchical clusters shown in Figure 1. (PDF 55 KB) [file 12859_2014_6670_MOESM1_ESM.pdf]

Confusion matrices

Here we give confusion matrices obtained from each of the classifiers. These matrices were used in creating the hierarchical clusters shown in figure 1. These matrices were obtained as a result of 5 times 20-fold cross-validation.

HT29 cells: Standard gentle boosting

Table 1

	AB	AD	AT	ACE	CN	LSC	LP	MP	M	PIP	PA	PHD	PMP	PP
**AB**	470	0	0	0	35	5	0	0	17	0	0	4	4	0
**AD**	1	514	10	3	9	0	0	0	1	1	0	5	6	5
**AT**	0	0	857	0	6	0	0	27	0	1	0	0	19	0
**ACE**	3	0	0	213	18	6	16	0	107	0	0	2	0	0
**CN**	38	22	3	3	775	24	5	16	31	0	2	6	0	0
**LSC**	1	0	2	1	15	815	0	0	114	0	32	25	0	0
**LP**	5	2	0	22	12	2	228	0	24	0	0	0	0	0
**MP**	0	0	2	0	3	0	0	2738	0	0	0	1	71	0
**M**	18	6	0	49	30	165	9	0	653	0	16	4	0	0
**PIP**	0	6	19	0	5	8	5	4	3	118	0	0	2	0
**PA**	0	0	0	0	0	80	0	0	35	0	170	10	0	0
**PHD**	4	0	1	0	0	11	0	0	1	0	0	1303	0	0
**PMP**	0	0	3	0	2	0	0	101	0	0	0	2	1607	10
**PP**	0	1	3	0	0	0	0	1	0	0	0	5	29	726

HT29 cells: SVM (linear)

Table 2

	AB	AD	AT	ACE	CN	LSC	LP	MP	M	PIP	PA	PHD	PMP	PP
**AB**	478	0	0	2	26	0	0	5	8	1	0	10	5	0
**AD**	2	528	10	0	0	0	0	5	0	0	0	5	0	5
**AT**	0	0	840	0	8	0	0	37	0	10	0	5	10	0
**ACE**	0	0	0	191	10	1	15	0	139	0	0	5	0	4
**CN**	36	38	1	13	742	40	10	15	23	0	0	7	0	0
**LSC**	0	0	0	0	10	793	0	0	144	0	33	25	0	0
**LP**	5	0	0	14	0	0	262	0	14	0	0	0	0	0
**MP**	0	0	0	0	0	0	0	2720	0	0	0	0	91	4
**M**	22	10	0	45	27	165	16	0	635	0	30	0	0	0
**PIP**	10	10	23	0	0	5	0	0	0	117	0	0	5	0
**PA**	0	0	0	0	5	87	0	0	30	0	163	10	0	0
**PHD**	9	0	0	5	0	16	0	0	2	0	0	1283	5	0
**PMP**	0	0	0	0	4	0	0	119	0	0	0	0	1592	10
**PP**	0	0	0	0	0	0	0	8	0	0	0	0	17	740

HT29 cells: SVM (RBF)

Table 3

	AB	AD	AT	ACE	CN	LSC	LP	MP	M	PIP	PA	PHD	PMP	PP
**AB**	483	0	2	0	22	0	0	0	10	0	0	16	2	0
**AD**	5	513	10	0	7	0	0	5	0	5	0	5	0	5
**AT**	0	0	856	0	0	0	0	24	0	15	0	0	15	0
**ACE**	0	0	0	202	15	5	20	0	113	0	0	10	0	0
**CN**	34	32	0	13	740	40	8	15	28	10	0	5	0	0
**LSC**	0	0	0	0	16	819	0	0	102	0	43	25	0	0
**LP**	5	0	0	15	0	0	261	0	14	0	0	0	0	0
**MP**	0	0	0	0	0	0	0	2730	0	0	0	0	81	4
**M**	7	5	0	59	28	174	10	0	639	0	23	5	0	0
**PIP**	10	5	20	1	5	0	0	0	2	122	0	0	5	0
**PA**	0	0	0	0	0	100	0	0	25	0	160	10	0	0
**PHD**	0	5	0	0	0	24	0	5	6	0	0	1280	0	0
**PMP**	0	0	5	0	0	0	0	85	0	0	0	0	1619	16
**PP**	0	0	5	0	0	0	0	5	0	0	0	0	20	735

HT29 cells: LDA

Table 4

	AB	AD	AT	ACE	CN	LSC	LP	MP	M	PIP	PA	PHD	PMP	PP
**AB**	440	0	0	13	32	1	1	0	31	0	2	15	0	0
**AD**	1	482	10	8	19	0	0	2	5	15	0	5	3	5
**AT**	0	4	800	0	10	0	0	55	0	31	0	0	10	0
**ACE**	2	0	0	226	8	0	17	0	107	0	0	0	5	0
**CN**	33	26	0	5	758	14	5	14	58	9	2	0	1	0
**LSC**	3	1	0	3	6	651	4	0	197	5	120	15	0	0
**LP**	0	0	0	27	0	0	237	0	26	5	0	0	0	0
**MP**	0	0	5	0	0	0	0	2726	0	0	0	0	84	0
**M**	8	10	0	79	9	143	8	0	653	0	40	0	0	0
**PIP**	0	9	20	14	1	0	0	0	3	118	0	0	5	0
**PA**	0	0	0	0	0	42	0	0	16	0	227	10	0	0
**PHD**	4	0	0	2	0	2	0	0	3	0	3	1301	0	5
**PMP**	0	0	9	0	0	0	0	105	0	0	0	0	1601	10
**PP**	0	0	5	5	0	0	0	7	0	0	0	0	29	719

HeLa cells: Standard gentle boosting

Table 5

	AF	BC	C	D	LA	MP	MB	N	P	Z
**AF**	571	148	6	7	22	0	1	62	33	0
**BC**	60	1148	33	10	3	27	0	163	101	5
**C**	12	64	1252	33	23	70	27	116	36	57
**D**	6	8	63	938	3	20	0	30	12	15
**LA**	10	9	12	2	1005	2	1	229	20	0
**MP**	15	36	64	5	7	733	0	19	32	19
**MB**	0	0	103	0	2	0	346	77	17	5
**N**	14	70	75	8	211	22	19	2160	126	5
**P**	38	143	47	16	39	31	9	181	1058	13
**Z**	0	0	96	20	0	10	5	1	11	342

HeLa cells: SVM (linear)

Table 6

	AF	BC	C	D	LA	MP	MB	N	P	Z
**AF**	634	110	0	3	9	0	5	69	20	0
**BC**	62	1113	27	10	5	25	0	202	101	5
**C**	10	73	1217	28	22	75	37	153	35	40
**D**	5	5	57	950	10	17	5	30	10	6
**LA**	15	1	5	0	1008	5	0	242	14	0
**MP**	20	29	21	5	0	785	0	27	27	16
**MB**	0	0	74	0	0	0	410	43	23	0
**N**	10	51	48	1	177	20	31	2277	90	5
**P**	26	120	33	14	49	30	5	186	1095	17
**Z**	0	0	90	21	0	10	0	5	10	349

HeLa cells: SVM (RBF)

Table 7

	AF	BC	C	D	LA	MP	MB	N	P	Z
**AF**	637	113	0	7	8	0	0	47	38	0
**BC**	60	1210	20	8	2	36	0	95	114	5
**C**	10	66	1333	32	16	42	29	69	39	54
**D**	5	6	58	962	10	19	5	13	10	7
**LA**	11	14	13	5	975	0	0	252	20	0
**MP**	15	30	26	9	0	786	0	19	25	20
**MB**	0	0	72	0	0	0	405	41	27	5
**N**	23	73	43	3	207	20	14	2207	115	5
**P**	25	141	26	19	45	29	11	160	1104	15
**Z**	0	0	87	16	0	0	0	0	10	372

HeLa cells: LDA

Table 8

	AF	BC	C	D	LA	MP	MB	N	P	Z
**AF**	654	111	0	0	5	5	0	55	20	0
**BC**	102	1095	25	5	11	45	5	158	99	5
**C**	20	60	1156	10	10	106	59	148	30	91
**D**	10	5	75	858	4	18	5	65	15	40
**LA**	20	0	1	0	1005	0	0	254	10	0
**MP**	27	18	10	0	0	807	0	15	30	23
**MB**	5	0	80	0	0	0	395	40	25	5
**N**	19	63	64	5	200	20	34	2233	67	5
**P**	35	126	40	5	44	30	10	193	1077	15
**Z**	0	0	75	5	0	21	2	0	10	372
